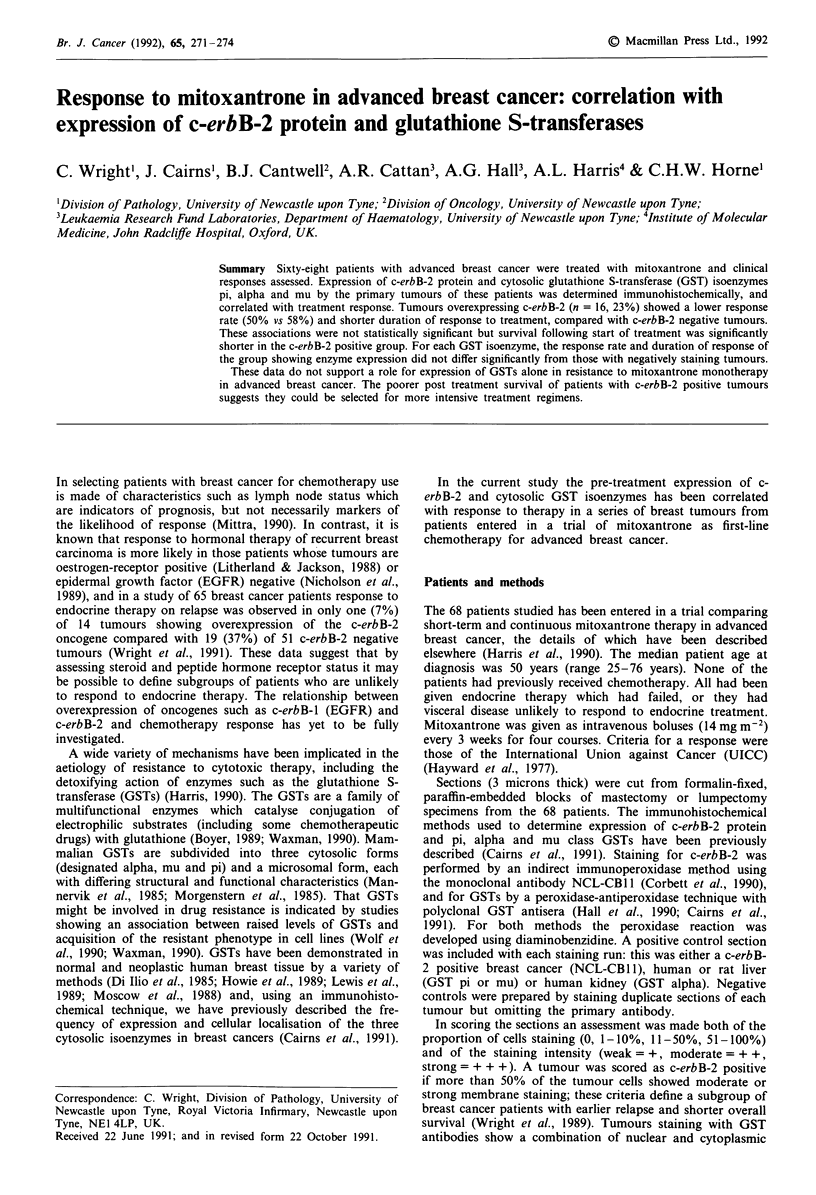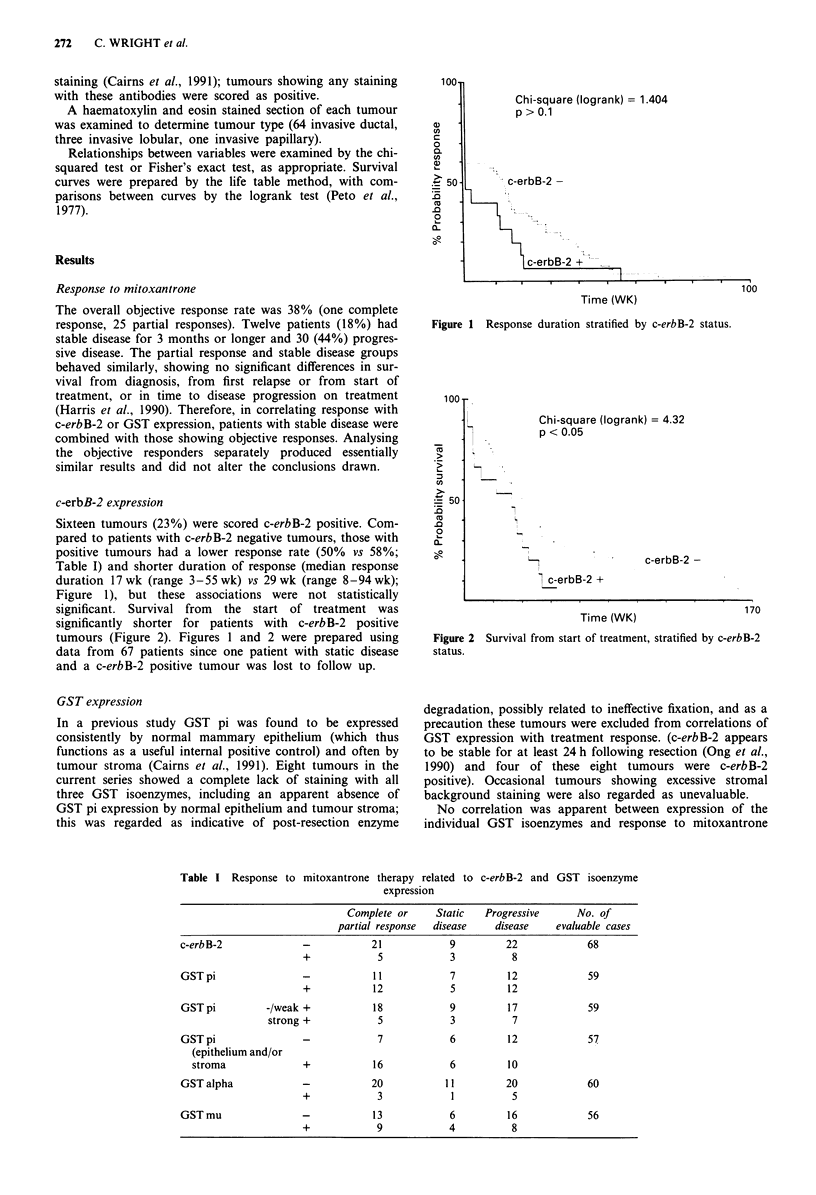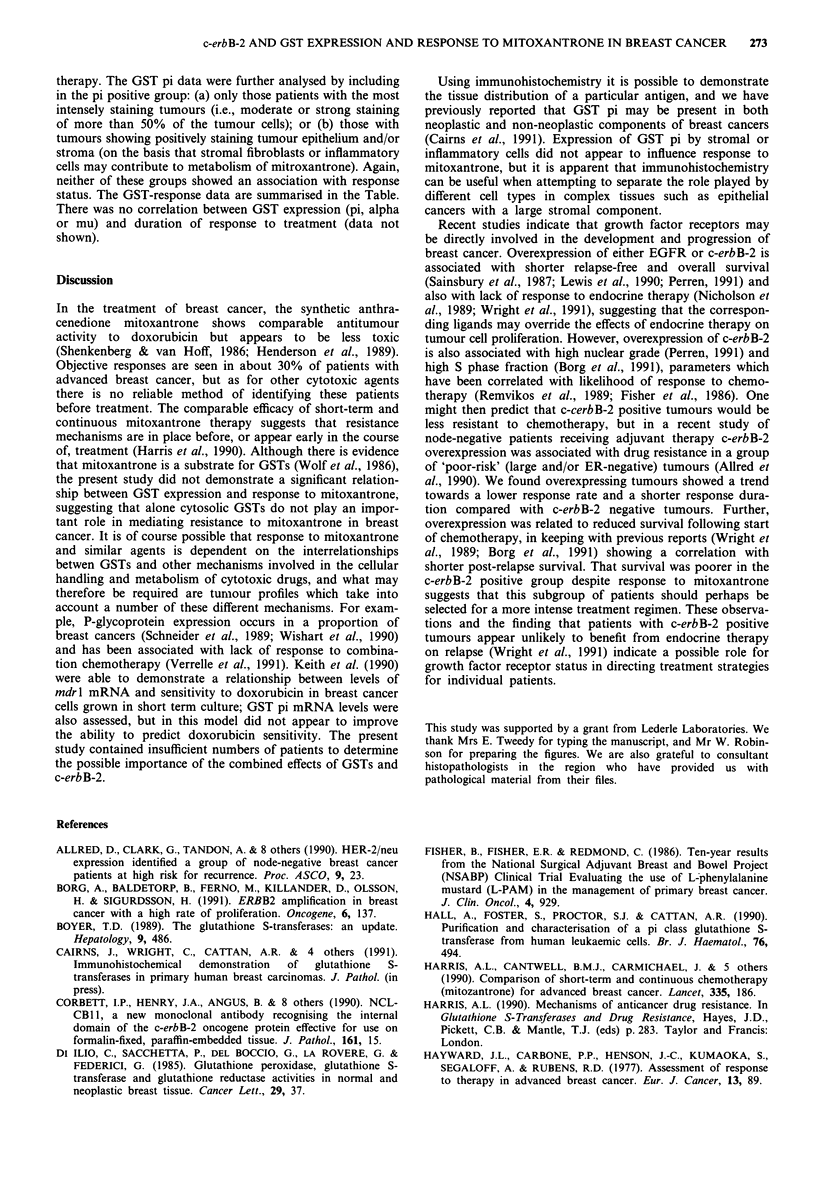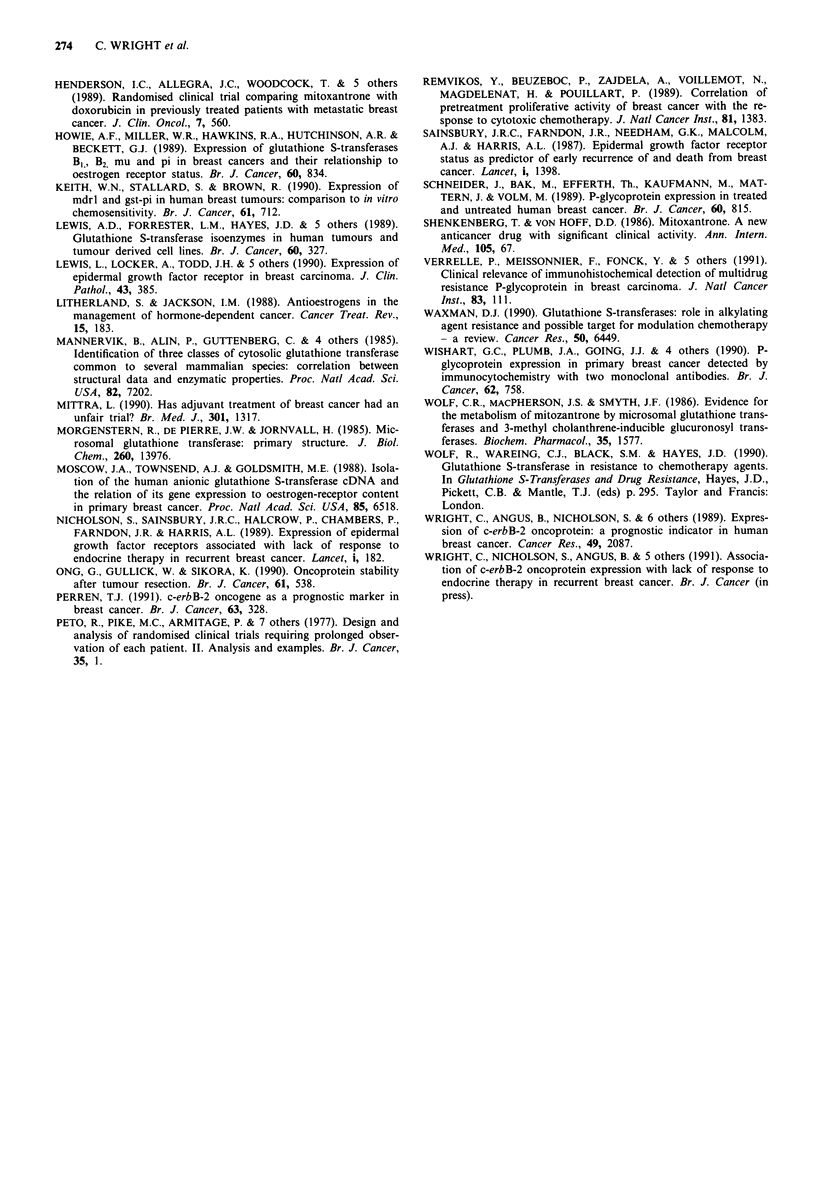# Response to mitoxantrone in advanced breast cancer: correlation with expression of c-erbB-2 protein and glutathione S-transferases.

**DOI:** 10.1038/bjc.1992.54

**Published:** 1992-02

**Authors:** C. Wright, J. Cairns, B. J. Cantwell, A. R. Cattan, A. G. Hall, A. L. Harris, C. H. Horne

**Affiliations:** Division of Pathology, University of Newcastle upon Tyne, UK.

## Abstract

Sixty-eight patients with advanced breast cancer were treated with mitoxantrone and clinical responses assessed. Expression of c-erbB-2 protein and cytosolic glutathione S-transferase (GST) isoenzymes pi, alpha and mu by the primary tumours of these patients was determined immunohistochemically, and correlated with treatment response. Tumours overexpressing c-erbB-2 (n = 16, 23%) showed a lower response rate (50% vs 58%) and shorter duration of response to treatment, compared with c-erbB-2 negative tumours. These associations were not statistically significant but survival following start of treatment was significantly shorter in the c-erbB-2 positive group. For each GST isoenzyme, the response rate and duration of response of the group showing enzyme expression did not differ significantly from those with negatively staining tumours. These data do not support a role for expression of GSTs alone in resistance to mitoxantrone monotherapy in advanced breast cancer. The poorer post treatment survival of patients with c-erbB-2 positive tumours suggests they could be selected for more intensive treatment regimens.


Br. J. Cancer (1992), 65, 271–274 © Macmillan Press Ltd., 1992

### Response to mitoxantrone in advanced breast cancer: correlation with expression of c-*erb*B-2 protein and glutathione S-transferases

C. Wright[1], J. Cairns[1], B.J. Cantwell[2], A.R. Cattan[3], A.G. Hall[3], A.L. Harris[4] & C.H.W. Horne[1]

[1]*Division of Pathology, University of Newcastle upon Tyne;* [2]*Division of Oncology, University of Newcastle upon Tyne;* [3]*Leukaemia Research Fund Laboratories, Department of Haematology, University of Newcastle upon Tyne;* [4]*Institute of Molecular Medicine, John Radcliffe Hospital, Oxford, UK.*

**Summary** Sixty-eight patients with advanced breast cancer were treated with mitoxantrone and clinical responses assessed. Expression of c-*erb*B-2 protein and cytosolic glutathione S-transferase (GST) isoenzymes pi, alpha and mu by the primary tumours of these patients was determined immunohistochemically, and correlated with treatment response. Tumours overexpressing c-*erb*B-2 ($n$ = 16, 23%) showed a lower response rate (50% *vs* 58%) and shorter duration of response to treatment, compared with c-*erb*B-2 negative tumours. These associations were not statistically significant but survival following start of treatment was significantly shorter in the c-*erb*B-2 positive group. For each GST isoenzyme, the response rate and duration of response of the group showing enzyme expression did not differ significantly from those with negatively staining tumours.

These data do not support a role for expression of GSTs alone in resistance to mitoxantrone monotherapy in advanced breast cancer. The poorer post treatment survival of patients with c-*erb*B-2 positive tumours suggests they could be selected for more intensive treatment regimens.

In selecting patients with breast cancer for chemotherapy use is made of characteristics such as lymph node status which are indicators of prognosis, but not necessarily markers of the likelihood of response (Mittra, 1990). In contrast, it is known that response to hormonal therapy of recurrent breast carcinoma is more likely in those patients whose tumours are oestrogen-receptor positive (Litherland & Jackson, 1988) or epidermal growth factor (EGFR) negative (Nicholson *et al.*, 1989), and in a study of 65 breast cancer patients response to endocrine therapy on relapse was observed in only one (7%) of 14 tumours showing overexpression of the c-*erb*B-2 oncogene compared with 19 (37%) of 51 c-*erb*B-2 negative tumours (Wright *et al.*, 1991). These data suggest that by assessing steroid and peptide hormone receptor status it may be possible to define subgroups of patients who are unlikely to respond to endocrine therapy. The relationship between overexpression of oncogenes such as c-*erb*B-1 (EGFR) and c-*erb*B-2 and chemotherapy response has yet to be fully investigated.

A wide variety of mechanisms have been implicated in the aetiology of resistance to cytotoxic therapy, including the detoxifying action of enzymes such as the glutathione S-transferase (GSTs) (Harris, 1990). The GSTs are a family of multifunctional enzymes which catalyse conjugation of electrophilic substrates (including some chemotherapeutic drugs) with glutathione (Boyer, 1989; Waxman, 1990). Mammalian GSTs are subdivided into three cytosolic forms (designated alpha, mu and pi) and a microsomal form, each with differing structural and functional characteristics (Mannervik *et al.*, 1985; Morgenstern *et al.*, 1985). That GSTs might be involved in drug resistance is indicated by studies showing an association between raised levels of GSTs and acquisition of the resistant phenotype in cell lines (Wolf *et al.*, 1990; Waxman, 1990). GSTs have been demonstrated in normal and neoplastic human breast tissue by a variety of methods (Di Ilio *et al.*, 1985; Howie *et al.*, 1989; Lewis *et al.*, 1989; Moscow *et al.*, 1988) and, using an immunohistochemical technique, we have previously described the frequency of expression and cellular localisation of the three cytosolic isoenzymes in breast cancers (Cairns *et al.*, 1991).

Correspondence: C. Wright, Division of Pathology, University of Newcastle upon Tyne, Royal Victoria Infirmary, Newcastle upon Tyne, NE1 4LP, UK.
Received 22 June 1991; and in revised form 22 October 1991.

In the current study the pre-treatment expression of c-*erb*B-2 and cytosolic GST isoenzymes has been correlated with response to therapy in a series of breast tumours from patients entered in a trial of mitoxantrone as first-line chemotherapy for advanced breast cancer.

### Patients and methods

The 68 patients studied has been entered in a trial comparing short-term and continuous mitoxantrone therapy in advanced breast cancer, the details of which have been described elsewhere (Harris *et al.*, 1990). The median patient age at diagnosis was 50 years (range 25–76 years). None of the patients had previously received chemotherapy. All had been given endocrine therapy which had failed, or they had visceral disease unlikely to respond to endocrine treatment. Mitoxantrone was given as intravenous boluses ($14\ mg\ m^{-2}$) every 3 weeks for four courses. Criteria for a response were those of the International Union against Cancer (UICC) (Hayward *et al.*, 1977).

Sections (3 microns thick) were cut from formalin-fixed, paraffin-embedded blocks of mastectomy or lumpectomy specimens from the 68 patients. The immunohistochemical methods used to determine expression of c-*erb*B-2 protein and pi, alpha and mu class GSTs have been previously described (Cairns *et al.*, 1991). Staining for c-*erb*B-2 was performed by an indirect immunoperoxidase method using the monoclonal antibody NCL-CB11 (Corbett *et al.*, 1990), and for GSTs by a peroxidase-antiperoxidase technique with polyclonal GST antisera (Hall *et al.*, 1990; Cairns *et al.*, 1991). For both methods the peroxidase reaction was developed using diaminobenzidine. A positive control section was included with each staining run: this was either a c-*erb*B-2 positive breast cancer (NCL-CB11), human or rat liver (GST pi or mu) or human kidney (GST alpha). Negative controls were prepared by staining duplicate sections of each tumour but omitting the primary antibody.

In scoring the sections an assessment was made both of the proportion of cells staining (0, 1–10%, 11–50%, 51–100%) and of the staining intensity (weak = +, moderate = + +, strong = + + +). A tumour was scored as c-*erb*B-2 positive if more than 50% of the tumour cells showed moderate or strong membrane staining; these criteria define a subgroup of breast cancer patients with earlier relapse and shorter overall survival (Wright *et al.*, 1989). Tumours staining with GST antibodies show a combination of nuclear and cytoplasmic staining (Cairns *et al.*, 1991); tumours showing any staining with these antibodies were scored as positive.

A haematoxylin and eosin stained section of each tumour was examined to determine tumour type (64 invasive ductal, three invasive lobular, one invasive papillary).

Relationships between variables were examined by the chi-squared test or Fisher's exact test, as appropriate. Survival curves were prepared by the life table method, with comparisons between curves by the logrank test (Peto *et al.*, 1977).

### Results

### *Response to mitoxantrone*

The overall objective response rate was 38% (one complete response, 25 partial responses). Twelve patients (18%) had stable disease for 3 months or longer and 30 (44%) progressive disease. The partial response and stable disease groups behaved similarly, showing no significant differences in survival from diagnosis, from first relapse or from start of treatment, or in time to disease progression on treatment (Harris *et al.*, 1990). Therefore, in correlating response with c-*erb*B-2 or GST expression, patients with stable disease were combined with those showing objective responses. Analysing the objective responders separately produced essentially similar results and did not alter the conclusions drawn.

### *c-*erb*B-2 expression*

Sixteen tumours (23%) were scored c-*erb*B-2 positive. Compared to patients with c-*erb*B-2 negative tumours, those with positive tumours had a lower response rate (50% *vs* 58%; Table I) and shorter duration of response (median response duration 17 wk (range 3–55 wk) *vs* 29 wk (range 8–94 wk); Figure 1), but these associations were not statistically significant. Survival from the start of treatment was significantly shorter for patients with c-*erb*B-2 positive tumours (Figure 2). Figures 1 and 2 were prepared using data from 67 patients since one patient with static disease and a c-*erb*B-2 positive tumour was lost to follow up.

**Figure 1** Response duration stratified by c-*erb*B-2 status.

**Figure 2** Survival from start of treatment, stratified by c-*erb*B-2 status.

### *GST expression*

In a previous study GST pi was found to be expressed consistently by normal mammary epithelium (which thus functions as a useful internal positive control) and often by tumour stroma (Cairns *et al.*, 1991). Eight tumours in the current series showed a complete lack of staining with all three GST isoenzymes, including an apparent absence of GST pi expression by normal epithelium and tumour stroma; this was regarded as indicative of post-resection enzyme degradation, possibly related to ineffective fixation, and as a precaution these tumours were excluded from correlations of GST expression with treatment response. (c-*erb*B-2 appears to be stable for at least 24 h following resection (Ong *et al.*, 1990) and four of these eight tumours were c-*erb*B-2 positive). Occasional tumours showing excessive stromal background staining were also regarded as unevaluable.

No correlation was apparent between expression of the individual GST isoenzymes and response to mitoxantrone

**Table I** Response to mitoxantrone therapy related to c-*erb*B-2 and GST isoenzyme expression

| | | *Complete or partial response* | *Static disease* | *Progressive disease* | *No. of evaluable cases* |
|---|---|---|---|---|---|
| c-*erb*B-2 | – | 21 | 9 | 22 | 68 |
| | + | 5 | 3 | 8 | |
| GST pi | – | 11 | 7 | 12 | 59 |
| | + | 12 | 5 | 12 | |
| GST pi | -/weak + | 18 | 9 | 17 | 59 |
| | strong + | 5 | 3 | 7 | |
| GST pi (epithelium and/or | – | 7 | 6 | 12 | 57 |
| stroma | + | 16 | 6 | 10 | |
| GST alpha | – | 20 | 11 | 20 | 60 |
| | + | 3 | 1 | 5 | |
| GST mu | – | 13 | 6 | 16 | 56 |
| | + | 9 | 4 | 8 | |

therapy. The GST pi data were further analysed by including in the pi positive group: (a) only those patients with the most intensely staining tumours (i.e., moderate or strong staining of more than 50% of the tumour cells); or (b) those with tumours showing positively staining tumour epithelium and/or stroma (on the basis that stromal fibroblasts or inflammatory cells may contribute to metabolism of mitroxantrone). Again, neither of these groups showed an association with response status. The GST-response data are summarised in the Table. There was no correlation between GST expression (pi, alpha or mu) and duration of response to treatment (data not shown).

### Discussion

In the treatment of breast cancer, the synthetic anthracenedione mitoxantrone shows comparable antitumour activity to doxorubicin but appears to be less toxic (Shenkenberg & van Hoff, 1986; Henderson *et al.*, 1989). Objective responses are seen in about 30% of patients with advanced breast cancer, but as for other cytotoxic agents there is no reliable method of identifying these patients before treatment. The comparable efficacy of short-term and continuous mitoxantrone therapy suggests that resistance mechanisms are in place before, or appear early in the course of, treatment (Harris *et al.*, 1990). Although there is evidence that mitoxantrone is a substrate for GSTs (Wolf *et al.*, 1986), the present study did not demonstrate a significant relationship between GST expression and response to mitoxantrone, suggesting that alone cytosolic GSTs do not play an important role in mediating resistance to mitoxantrone in breast cancer. It is of course possible that response to mitoxantrone and similar agents is dependent on the interrelationships betwen GSTs and other mechanisms involved in the cellular handling and metabolism of cytotoxic drugs, and what may therefore be required are tumour profiles which take into account a number of these different mechanisms. For example, P-glycoprotein expression occurs in a proportion of breast cancers (Schneider *et al.*, 1989; Wishart *et al.*, 1990) and has been associated with lack of response to combination chemotherapy (Verrelle *et al.*, 1991). Keith *et al.* (1990) were able to demonstrate a relationship between levels of *mdr*1 mRNA and sensitivity to doxorubicin in breast cancer cells grown in short term culture; GST pi mRNA levels were also assessed, but in this model did not appear to improve the ability to predict doxorubicin sensitivity. The present study contained insufficient numbers of patients to determine the possible importance of the combined effects of GSTs and c-*erb*B-2.

Using immunohistochemistry it is possible to demonstrate the tissue distribution of a particular antigen, and we have previously reported that GST pi may be present in both neoplastic and non-neoplastic components of breast cancers (Cairns *et al.*, 1991). Expression of GST pi by stromal or inflammatory cells did not appear to influence response to mitoxantrone, but it is apparent that immunohistochemistry can be useful when attempting to separate the role played by different cell types in complex tissues such as epithelial cancers with a large stromal component.

Recent studies indicate that growth factor receptors may be directly involved in the development and progression of breast cancer. Overexpression of either EGFR or c-*erb*B-2 is associated with shorter relapse-free and overall survival (Sainsbury *et al.*, 1987; Lewis *et al.*, 1990; Perren, 1991) and also with lack of response to endocrine therapy (Nicholson *et al.*, 1989; Wright *et al.*, 1991), suggesting that the corresponding ligands may override the effects of endocrine therapy on tumour cell proliferation. However, overexpression of c-*erb*B-2 is also associated with high nuclear grade (Perren, 1991) and high S phase fraction (Borg *et al.*, 1991), parameters which have been correlated with likelihood of response to chemotherapy (Remvikos *et al.*, 1989; Fisher *et al.*, 1986). One might then predict that c-*cerb*B-2 positive tumours would be less resistant to chemotherapy, but in a recent study of node-negative patients receiving adjuvant therapy c-*erb*B-2 overexpression was associated with drug resistance in a group of 'poor-risk' (large and/or ER-negative) tumours (Allred *et al.*, 1990). We found overexpressing tumours showed a trend towards a lower response rate and a shorter response duration compared with c-*erb*B-2 negative tumours. Further, overexpression was related to reduced survival following start of chemotherapy, in keeping with previous reports (Wright *et al.*, 1989; Borg *et al.*, 1991) showing a correlation with shorter post-relapse survival. That survival was poorer in the c-*erb*B-2 positive group despite response to mitoxantrone suggests that this subgroup of patients should perhaps be selected for a more intense treatment regimen. These observations and the finding that patients with c-*erb*B-2 positive tumours appear unlikely to benefit from endocrine therapy on relapse (Wright *et al.*, 1991) indicate a possible role for growth factor receptor status in directing treatment strategies for individual patients.

This study was supported by a grant from Lederle Laboratories. We thank Mrs E. Tweedy for typing the manuscript, and Mr W. Robinson for preparing the figures. We are also grateful to consultant histopathologists in the region who have provided us with pathological material from their files.

### References

ALLRED, D., CLARK, G., TANDON, A. & 8 others (1990). HER-2/neu expression identified a group of node-negative breast cancer patients at high risk for recurrence. *Proc. ASCO*, **9**, 23.

BORG, A., BALDETORP, B., FERNO, M., KILLANDER, D., OLSSON, H. & SIGURDSSON, H. (1991). *ERB*B2 amplification in breast cancer with a high rate of proliferation. *Oncogene*, **6**, 137.

BOYER, T.D. (1989). The glutathione S-transferases: an update. *Hepatology*, **9**, 486.

CAIRNS, J., WRIGHT, C., CATTAN, A.R. & 4 others (1991). Immunohistochemical demonstration of glutathione S-transferases in primary human breast carcinomas. *J. Pathol.* (in press).

CORBETT, I.P., HENRY, J.A., ANGUS, B. & 8 others (1990). NCL-CB11, a new monoclonal antibody recognising the internal domain of the c-*erb*B-2 oncogene protein effective for use on formalin-fixed, paraffin-embedded tissue. *J. Pathol.*, **161**, 15.

DI ILIO, C., SACCHETTA, P., DEL BOCCIO, G., LA ROVERE, G. & FEDERICI, G. (1985). Glutathione peroxidase, glutathione S-transferase and glutathione reductase activities in normal and neoplastic breast tissue. *Cancer Lett.*, **29**, 37.

FISHER, B., FISHER, E.R. & REDMOND, C. (1986). Ten-year results from the National Surgical Adjuvant Breast and Bowel Project (NSABP) Clinical Trial Evaluating the use of L-phenylalanine mustard (L-PAM) in the management of primary breast cancer. *J. Clin. Oncol.*, **4**, 929.

HALL, A., FOSTER, S., PROCTOR, S.J. & CATTAN, A.R. (1990). Purification and characterisation of a pi class glutathione S-transferase from human leukaemic cells. *Br. J. Haematol.*, **76**, 494.

HARRIS, A.L., CANTWELL, B.M.J., CARMICHAEL, J. & 5 others (1990). Comparison of short-term and continuous chemotherapy (mitozantrone) for advanced breast cancer. *Lancet*, **335**, 186.

HARRIS, A.L. (1990). Mechanisms of anticancer drug resistance. In *Glutathione S-Transferases and Drug Resistance*, Hayes, J.D., Pickett, C.B. & Mantle, T.J. (eds) p. 283. Taylor and Francis: London.

HAYWARD, J.L., CARBONE, P.P., HENSON, J.-C., KUMAOKA, S., SEGALOFF, A. & RUBENS, R.D. (1977). Assessment of response to therapy in advanced breast cancer. *Eur. J. Cancer*, **13**, 89.

HENDERSON, I.C., ALLEGRA, J.C., WOODCOCK, T. & 5 others (1989). Randomised clinical trial comparing mitoxantrone with doxorubicin in previously treated patients with metastatic breast cancer. *J. Clin. Oncol.*, **7**, 560.

HOWIE, A.F., MILLER, W.R., HAWKINS, R.A., HUTCHINSON, A.R. & BECKETT, G.J. (1989). Expression of glutathione S-transferases $B_1$, $B_2$, mu and pi in breast cancers and their relationship to oestrogen receptor status. *Br. J. Cancer*, **60**, 834.

KEITH, W.N., STALLARD, S. & BROWN, R. (1990). Expression of mdr1 and gst-pi in human breast tumours: comparison to *in vitro* chemosensitivity. *Br. J. Cancer*, **61**, 712.

LEWIS, A.D., FORRESTER, L.M., HAYES, J.D. & 5 others (1989). Glutathione S-transferase isoenzymes in human tumours and tumour derived cell lines. *Br. J. Cancer*, **60**, 327.

LEWIS, L., LOCKER, A., TODD, J.H. & 5 others (1990). Expression of epidermal growth factor receptor in breast carcinoma. *J. Clin. Pathol.*, **43**, 385.

LITHERLAND, S. & JACKSON, I.M. (1988). Antioestrogens in the management of hormone-dependent cancer. *Cancer Treat. Rev.*, **15**, 183.

MANNERVIK, B., ALIN, P., GUTTENBERG, C. & 4 others (1985). Identification of three classes of cytosolic glutathione transferase common to several mammalian species: correlation between structural data and enzymatic properties. *Proc. Natl Acad. Sci. USA*, **82**, 7202.

MITTRA, L. (1990). Has adjuvant treatment of breast cancer had an unfair trial? *Br. Med. J.*, **301**, 1317.

MORGENSTERN, R., DE PIERRE, J.W. & JORNVALL, H. (1985). Microsomal glutathione transferase: primary structure. *J. Biol. Chem.*, **260**, 13976.

MOSCOW, J.A., TOWNSEND, A.J. & GOLDSMITH, M.E. (1988). Isolation of the human anionic glutathione S-transferase cDNA and the relation of its gene expression to oestrogen-receptor content in primary breast cancer. *Proc. Natl Acad. Sci. USA*, **85**, 6518.

NICHOLSON, S., SAINSBURY, J.R.C., HALCROW, P., CHAMBERS, P., FARNDON, J.R. & HARRIS, A.L. (1989). Expression of epidermal growth factor receptors associated with lack of response to endocrine therapy in recurrent breast cancer. *Lancet*, **i**, 182.

ONG, G., GULLICK, W. & SIKORA, K. (1990). Oncoprotein stability after tumour resection. *Br. J. Cancer*, **61**, 538.

PERREN, T.J. (1991). c-*erb*B-2 oncogene as a prognostic marker in breast cancer. *Br. J. Cancer*, **63**, 328.

PETO, R., PIKE, M.C., ARMITAGE, P. & 7 others (1977). Design and analysis of randomised clinical trials requiring prolonged observation of each patient. II. Analysis and examples. *Br. J. Cancer*, **35**, 1.

REMVIKOS, Y., BEUZEBOC, P., ZAJDELA, A., VOILLEMOT, N., MAGDELENAT, H. & POUILLART, P. (1989). Correlation of pretreatment proliferative activity of breast cancer with the response to cytotoxic chemotherapy. *J. Natl Cancer Inst.*, **81**, 1383.

SAINSBURY, J.R.C., FARNDON, J.R., NEEDHAM, G.K., MALCOLM, A.J. & HARRIS, A.L. (1987). Epidermal growth factor receptor status as predictor of early recurrence of and death from breast cancer. *Lancet*, **i**, 1398.

SCHNEIDER, J., BAK, M., EFFERTH, Th., KAUFMANN, M., MATTERN, J. & VOLM, M. (1989). P-glycoprotein expression in treated and untreated human breast cancer. *Br. J. Cancer*, **60**, 815.

SHENKENBERG, T. & VON HOFF, D.D. (1986). Mitoxantrone. A new anticancer drug with significant clinical activity. *Ann. Intern. Med.*, **105**, 67.

VERRELLE, P., MEISSONNIER, F., FONCK, Y. & 5 others (1991). Clinical relevance of immunohistochemical detection of multidrug resistance P-glycoprotein in breast carcinoma. *J. Natl Cancer Inst.*, **83**, 111.

WAXMAN, D.J. (1990). Glutathione S-transferases: role in alkylating agent resistance and possible target for modulation chemotherapy – a review. *Cancer Res.*, **50**, 6449.

WISHART, G.C., PLUMB, J.A., GOING, J.J. & 4 others (1990). P-glycoprotein expression in primary breast cancer detected by immunocytochemistry with two monoclonal antibodies. *Br. J. Cancer*, **62**, 758.

WOLF, C.R., MACPHERSON, J.S. & SMYTH, J.F. (1986). Evidence for the metabolism of mitozantrone by microsomal glutathione transferases and 3-methyl cholanthrene-inducible glucuronosyl transferases. *Biochem. Pharmacol.*, **35**, 1577.

WOLF, R., WAREING, C.J., BLACK, S.M. & HAYES, J.D. (1990). Glutathione S-transferase in resistance to chemotherapy agents. In *Glutathione S-Transferases and Drug Resistance*, Hayes, J.D., Pickett, C.B. & Mantle, T.J. (eds) p. 295. Taylor and Francis: London.

WRIGHT, C., ANGUS, B., NICHOLSON, S. & 6 others (1989). Expression of c-*erb*B-2 oncoprotein: a prognostic indicator in human breast cancer. *Cancer Res.*, **49**, 2087.

WRIGHT, C., NICHOLSON, S., ANGUS, B. & 5 others (1991). Association of c-*erb*B-2 oncoprotein expression with lack of response to endocrine therapy in recurrent breast cancer. *Br. J. Cancer* (in press).